# Exploring the Use of a Web-Based Menu Planning Tool in Childcare Services: Qualitative Cross-sectional Survey Study

**DOI:** 10.2196/35553

**Published:** 2022-07-18

**Authors:** Jessica V Kempler, Penelope Love, Kristy A Bolton, Margaret Rozman, Alison C Spence

**Affiliations:** 1 School of Exercise and Nutrition Sciences Deakin University Geelong Australia; 2 Institute for Physical Activity and Nutrition (IPAN) Deakin University Geelong Australia; 3 Healthy Eating Advisory Service Nutrition Australia Victorian Division Docklands Australia

**Keywords:** child care, preschool, early childhood services, child nutrition, menu planning, healthy eating, web-based systems, web-based tool, internet-based intervention, user experience

## Abstract

**Background:**

Early childhood is a critical period for supporting the development of healthy eating habits, which may affect lifelong health. Childcare services are important settings for promoting early childhood nutrition; however, food provision in childcare frequently does not align with dietary guidelines. Web-based menu planning tools are well suited to support healthy food provision in childcare, although little is known about their use. Research is needed to understand how web-based menu planning tools are used in the childcare setting and how they can effectively support healthy menu planning and food provision for children in childcare.

**Objective:**

We aimed to explore the use of a web-based menu planning tool called *FoodChecker*, which is available to childcare services in Victoria, Australia. We also aimed to gain insights and perspectives from childcare staff involved in menu planning about their use of the tool to plan healthy menus and guide healthy food provision for children.

**Methods:**

We conducted a qualitative descriptive study using a cross-sectional web-based survey completed by the staff involved in menu planning in childcare services. Thematic analysis was performed using NVivo software. Emergent themes were mapped against constructs of the Technology Acceptance Model regarding perceived usefulness, perceived ease of use, and external variables influencing perceptions and use.

**Results:**

The participants included 30 cooks and 34 directors from 53 childcare services. Participants perceived the web-based menu planning tool as useful for supporting child nutrition and health, improving organizational processes, and aiding the menu planner role. Perceptions regarding ease of use were mixed. External variables influencing perceptions and use included awareness of the tool, perceived need, time, resources, organizational support, and the food budget. Participants made recommendations to improve the tool, particularly the need to integrate functionality to make it easier and faster to use or to include more links to resources to support healthy menu planning.

**Conclusions:**

The web-based menu planning tool was perceived as useful for cooks and directors in childcare services. Areas for improvement were identified; for example, the need for integrated digital features to make the tool easier and faster to use. As the first qualitative study to explore childcare staff experiences with a web-based menu planning tool, these findings inform future research and development of such tools to aid scalable and sustainable support for healthier food provision in the childcare sector.

## Introduction

### Background

Early childhood (commonly defined as 0-5 years) is a critical period for supporting the development of healthy eating habits that may track into later life [1]. An unhealthy diet during childhood is associated with both undernutrition and overweight and obesity [2]. As such, an unhealthy diet is a risk factor for nutrient deficiencies, impaired growth and development, and adverse chronic disease outcomes that can influence lifelong health [2-4]. Internationally, health [5] and government [6,7] authorities have established dietary guidelines outlining the types and amounts of foods children and adults are recommended to eat to support good health. However, population surveys demonstrate that globally, compliance with dietary guidelines is low across age groups and that children’s diets are suboptimal (eg, diets low in vegetables and high in energy-dense, nutrient-poor foods are prevalent) [8-11].

Setting-based health promotion, where health is created and lived by people within the settings of their everyday life [12], is widely advocated as evidence-based best practice [13]. Early childhood education and care settings have been identified in systematic reviews as opportune places to promote early childhood nutrition [14,15]. Long day care or center-based care (herein referred to as childcare) is the most common form of early childhood education and care setting in Australia, with almost 800,000 children attending for an average of 30.5 hours (approximately 3 days) per week [16]. Similarly high patterns of childcare attendance are observed across other high-income countries, for example, in European countries [17], the United States [18], and the United Kingdom [19], reflecting changes in family workforce patterns, including increased female participation and shared caring responsibilities [20].

In Australia, half of childcare services operate as private, for-profit organizations, while 35% are private and not-for-profit. The remainder are managed by state or local governments (11%) or nongovernment schools (4%) [21]. Families accessing childcare services are supported by a means-tested national government subsidy, whereby families with lower income are eligible for a greater subsidy amount [22]. However, this does not guarantee affordability for everyone, and access to childcare is unequally distributed across Australia, with regional, remote, and disadvantaged areas more likely to experience low provision or absence of childcare [23].

Childcare services commonly provide meals and snacks for attending children, contributing up to two-thirds of their daily food intake [24]. As such, they have an important opportunity to support early childhood nutrition, and fundamental to this is planning a healthy childcare menu [25]. Recognizing this, leading health [26-28] and childcare [29,30] authorities around the world have established recommendations for healthy menu planning and food provision in the childcare setting. For example, the World Health Organization Commission on Ending Childhood Obesity advocates mandatory childcare nutrition standards [26], and the Australian National Quality Standard [29] requires childcare services to ensure “healthy eating... [is] promoted and appropriate for each child” (Element 2.1.3). However, a broad, international evidence base indicates that childcare menus do not meet dietary guidelines and are suboptimal for both food [25,31-33] and nutrient [34,35] provision.

Several barriers to healthy food provision in childcare have been identified in the literature, including insufficient menu planning tools and support resources, lack of time, and limited nutrition and dietary guideline knowledge [36]. Although limited in number, small randomized controlled trials (RCTs) have shown that some intervention strategies, including menu auditing and feedback [37,38], provision of menu planning resources [37-39], and expert implementation support [37,39], can improve childcare food provision. However, implementation models have traditionally relied on in-person support [40] and ongoing resourcing [41], limiting the scalability and sustainability of intervention strategies to date.

Web-based menu planning tools are emerging as a novel strategy for improving childcare food provision [42,43]. Given that almost all childcare services have access to computers and the internet [44], web-based tools may provide a mechanism for delivering scalable and sustainable menu planning support across the childcare sector, including in geographically dispersed locations. Such tools can be embedded into existing web-based childcare management systems [43] and completed at a time, location, and pace convenient for end users, with modest financial and staff resourcing requirements compared with other mechanisms [15]. Using digitalized systems, web-based menu planning tools can integrate user-engagement features, such as automated calculations of food groups on menus, comparisons with dietary guidelines, provision of instant feedback reports, and direct links to easily accessible and relevant web-based support resources [42,43].

To our knowledge, only two published RCTs have considered the impact of childcare programs that incorporate web-based tools to support healthy menu planning and food provision: (1) a pilot RCT (n=31) of the *Go-NAPSACC* (Nutrition and Physical Activity Self-Assessment for Child Care) program in the United States [42] and (2) an Australian RCT (n=54) of the *feedAustralia* menu planning tool in the state of New South Wales [43]. Although both tools were shown to improve healthy food choices on childcare menus, neither resulted in significant increases in menu compliance with sector food provision guidelines [42,43]. Authors from both studies called for future research to identify factors that influence the implementation of web-based menu planning tools in the childcare setting and exploration of strategies to inform their widespread use across the sector [42,43].

The implementation and effectiveness of web-based health promotion tools in achieving public health impact is largely determined by end-user engagement [45]. In the childcare setting, users of web-based menu planning tools are most likely to be staff members who plan, prepare, and provide food for children—namely childcare cooks and directors [25,46]. However, little is known about how these users engage with web-based menu planning tools to support healthy food provision. From the limited evidence base, user acceptance of such programs is reportedly high, although studies to date have considered feedback from directors only [42,43] or captured only quantitative data [43]. This indicates that existing evidence may not reflect the nuanced perspectives of all stakeholders, including cooks and directors, who are likely to use web-based menu planning programs in practice.

**Figure 1:**
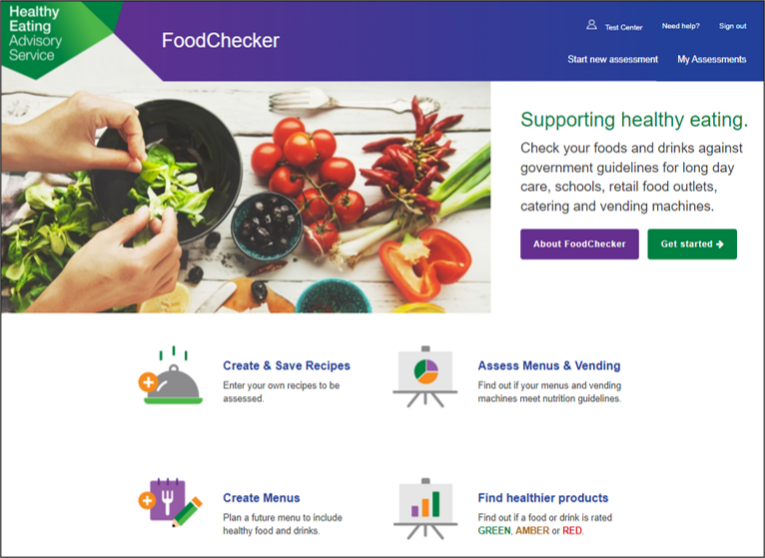
Screenshot of the *FoodChecker* homepage showing available services [47].

**Figure 2:**
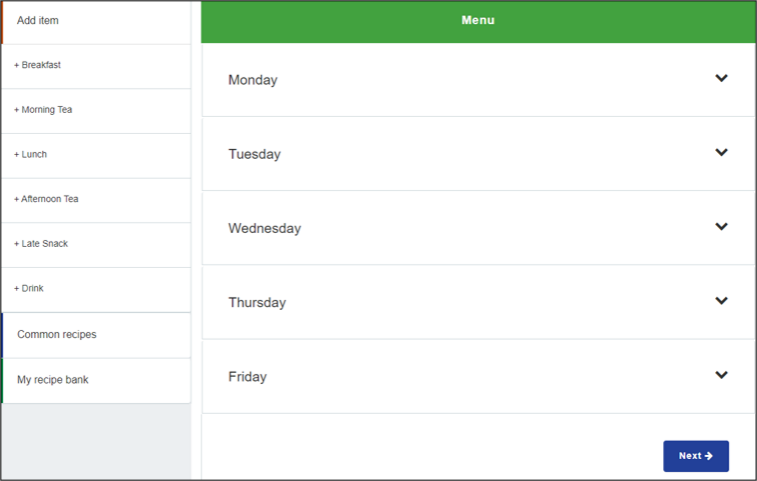
Screenshot of the *FoodChecker* menu data input template [47].

**Figure 3:**
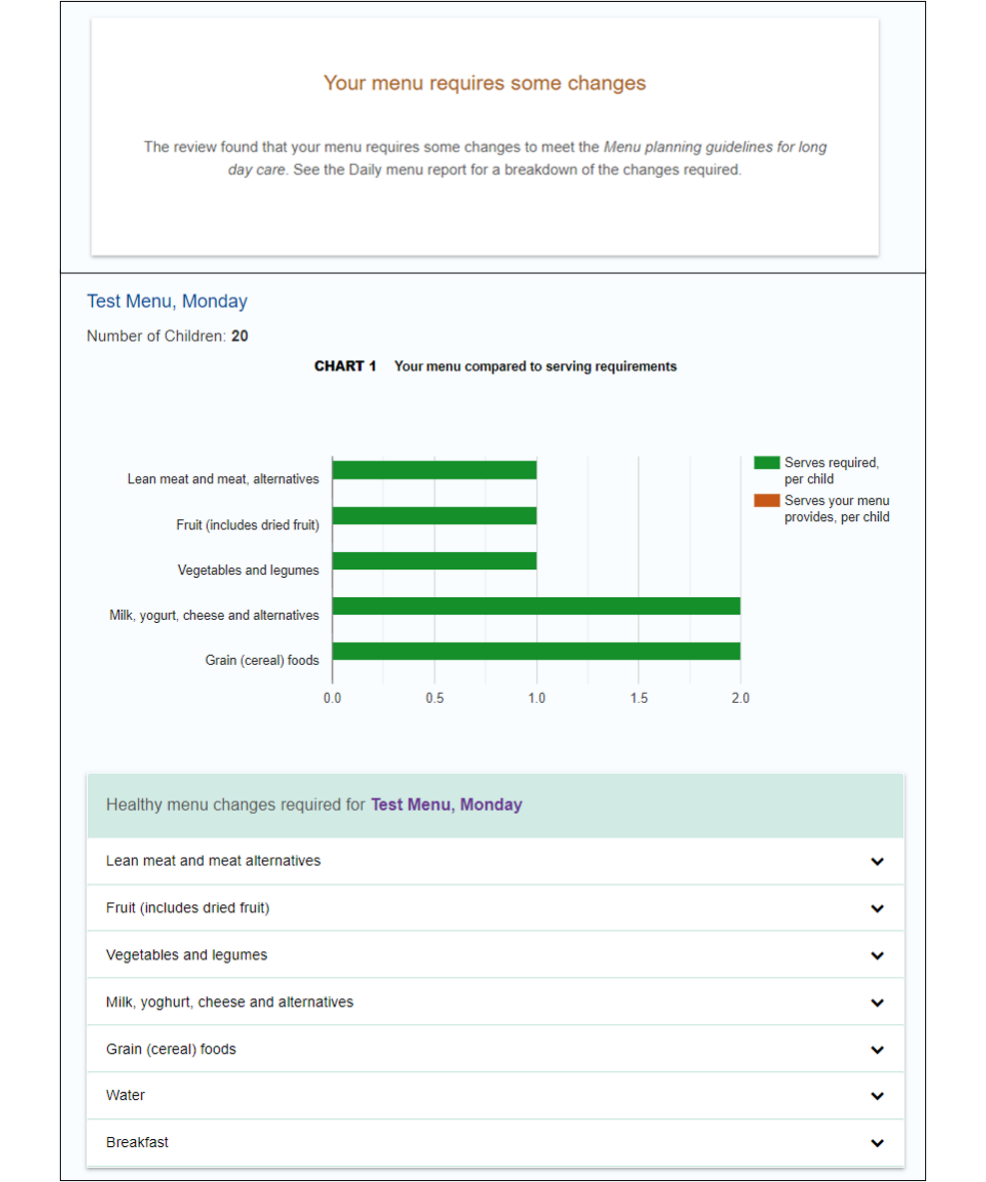
Screenshot of a sample automated report of menu alignment with dietary guidelines [47].

In the Australian state of Victoria, the Victorian government has invested in the development and implementation of a web-based menu planning tool called *FoodChecker* [47]. Delivered by Nutrition Australia Victorian Division (NAV), *FoodChecker* is freely available to all Victorian childcare services to support menu alignment with sector dietary recommendations [48]. *FoodChecker* has been used by a third of Victorian childcare services since its inception in 2017 (NAV Program Manager, personal communication, December 14, 2021). The flow of the *FoodChecker* website, including the home page, menu data input template, and a sample automated report of menu alignment with dietary guidelines, is shown in Figures 1-3 [47]. The rollout of *FoodChecker* provides an opportunity to explore the use of web-based menu planning tools for providing equitable, scalable, and sustainable menu planning support in the childcare sector.

### Objectives

Despite the potential of web-based menu planning tools to improve childcare food provision, to date, there are no published qualitative studies on the ways menu planning staff have engaged with these tools. The primary aim of this study was to explore the use of the web-based menu planning tool *FoodChecker* in Victorian childcare services. In particular, we aimed to gain insights and perspectives from menu planning staff members, in their own words, about their use of the web-based tool to plan healthy menus and guide healthy food provision for children in childcare.

## Methods

### Ethics Approval

This study was part of a broader research project on healthy eating and physical activity in childcare, with ethics approval from the Deakin University Human Ethics Advisory Group (HEAG-H91_2021). All participants provided voluntary and informed consent to participate and received an Aus $20 (US $13.94) gift card in appreciation of their time.

### Design and Setting

A qualitative descriptive study was conducted to explore participants’ perspectives about their use of a web-based menu planning tool in the childcare setting. Although all research team members held nutrition qualifications, they sought to learn from the experience and expertise of the childcare staff. The researchers held an ontological position that embraced subjectivity, focusing on participants’ personal experiences, insights, and opinions as opposed to seeking an *absolute truth*. The methods and results of this study were reported in accordance with the Standards for Reporting Qualitative Research checklist [49].

A cross-sectional survey of childcare staff members involved in planning childcare menus was conducted between July and September 2021 in Victoria, Australia. The survey explored the use of the web-based menu planning tool *FoodChecker*. Participant perceptions of *FoodChecker* were captured using a web-based qualitative survey, a data collection method recognized as beneficial for harnessing nuanced accounts of participant experiences within the qualitative descriptive paradigm [50].

### Participants

Childcare services were identified from the Australian Children’s Education and Care Quality Authority National Register [51] in July 2021. Eligible services were required to (1) be located in Victoria, Australia; (2) be open for at least 8 hours each weekday; (3) operate for at least 48 weeks annually; and (4) prepare and provide lunch, morning tea, and afternoon tea for attending children on each weekday. Services that did not provide food for children (eg, where meals were provided by parents) were ineligible because of differing meal planning requirements and because these represent a minority of childcare services in Victoria [52]. As childcare cooks and directors frequently share menu planning responsibilities [25,46], data were collected from both staff groups. A target sample size was not predetermined because of the inductive nature of the investigation and the desire to capture as broad a range of responses as possible from those with experiential expertise in childcare menu planning. Given that there are no published data on the proportion of childcare services in Victoria that provide food to children, the size of the target population was unknown. As such, data collection continued until no further responses were received.

### Recruitment

An email invitation was sent to directors of all Victorian childcare services on the Australian Children’s Education and Care Quality Authority National Register in July 2021 (N=1726) with a link to a voluntary self-administered *director survey* on the secure REDCap (Research Electronic Data Capture; Vanderbilt University) platform [53]. Directors providing consent responded to a screening question (within the *director survey*) regarding *FoodChecker* use (yes, no, or unsure). Directors who nominated that *FoodChecker* was or may have been used at their service were sent a link to a *FoodChecker survey*, which included individual consent. This could be forwarded to the cook responsible for planning the service’s menu or completed by the director if they were involved in menu planning. One reminder email was sent to directors who did not respond to the initial recruitment email after 2 weeks. To maximize cooks’ participation in the *FoodChecker survey*, the study was advertised to cooks in September 2021 via a post on a social media webpage commonly accessed by the target population.

### Data Collection and Measures

#### Childcare Service and Participant Characteristics

Similar to previous research within the Australian childcare setting [43], participants reported their childcare service postcode and type of management (private or community), as well as their role in the service, years of employment, educational attainment, and whether they had received nutrition training. Participants also reported whether their service had ever used *FoodChecker* for menu planning (yes, no, or unsure).

#### *FoodChecker* Survey Design

As this is the first study, to the best of our knowledge, focusing on the qualitative exploration of a web-based childcare menu planning tool, a set of questions about *FoodChecker* use was purpose-designed by the research team. Previous international studies on user experiences with digital health tools have identified the need to capture information in the domains of user attitudes, experiences and expectations, and resultant changes in confidence, learning, and behavior [54,55]. To ensure that the study needs were addressed, additional domains were included to capture information about the frequency and purpose of use of *FoodChecker* and barriers and enablers influencing use (Table 1).

Topic-based qualitative questions were designed to be open and as succinct, clear, and unambiguous as possible, using the guidance for designing qualitative survey questions provided by Braun et al [50]. To contribute to internal generalizability and to support the interpretation of findings within the qualitative analysis [56], 5 quantitative questions using a nominal (yes or no) scale were added. Questions were then tested for face validity by 7 researchers (including JVK, ACS, PL, and KAB) with expertise in early childhood nutrition, 3 NAV staff members (including MR), 1 user experience design professional, and 1 previous childcare cook, with feedback incorporated into the final survey questions. Readability scores for the final set of *FoodChecker* questions (n=16 questions; Table 1) were 69.8 on the Flesch Reading Ease Test (desirable range 60-70) and 5.9 on the Flesch-Kincaid Grade Level Test, indicating that the content could likely be understood by a person approaching sixth grade in the United States [57].

The *FoodChecker survey* included the complete set of *FoodChecker* questions. Four of these questions were included in the *director survey*. This approach preempted the expectation that most participants responding to the *FoodChecker survey* would be cooks but that it was also important to seek insights from directors who often play a role in menu planning [25,46]. For both surveys, the number of questions included was within the range of 4 to 16, which is commonly observed in the literature for qualitative survey analyses focusing on lived experiences [50].

**Table 1:** Domains and questions in the *FoodChecker* question set^a^.

Domain	Question
Frequency of use	Q1. How often do you use *FoodChecker*?
Purpose of use	Q2a. Please briefly state why you use or have used *FoodChecker*.^b^ or Q2b. Please explain why your center does not use *FoodChecker* for menu planning.^b^
User experiences	Q3. What is the first thing that comes to mind about your experience with using *FoodChecker*?
User attitudes	Q4. What do you like the most about using *FoodChecker*? Q5. What do you like the least about using *FoodChecker*? Q6. Do you think that online menu planning tools like *FoodChecker* are useful for your role? (yes/no) Please tell us why/why not. Q7. Do you think that online menu planning tools like *FoodChecker* are useful for childcare centers? (yes/no) Please tell us why/why not.^b^
Enablers to use	Q8. Have you accessed any support to help you use *FoodChecker*? Q9. What organizational support do you receive (if any) to use *FoodChecker*?
Barriers to use	Q10. What challenges do you face (if any) regarding the use of *FoodChecker*?
Changes in confidence, learning and behavior	Q11. What do you think has changed for you or your center as a result of using *FoodChecker*?^b^ Q12. As a result of using *FoodChecker*, has your confidence about planning healthy menus improved? (yes/no) Q13. As a result of using *FoodChecker*, have you learnt something? (yes/no) What have you learnt? Q14. As a result of using *FoodChecker*, has your center's menu changed? (yes/no) What has changed?
User expectations	Q15. If *FoodChecker* was being updated, is there anything that you would like to see in an “ideal” online menu planning tool?
Other	Q16. Is there anything else that you think is important for us to know about *FoodChecker*?

^a^All questions were included in the *FoodChecker survey*.

^b^Questions included in *director survey* with the additional question, “Who has used *FoodChecker* in your center?”

### Data Analysis

Survey response files were downloaded from REDCap, deidentified, and uploaded to NVivo (version 20; QSR International), a secure web-based data analysis platform [58].

#### Statistical Analysis

Postcode to remoteness area matching using the Australian Statistical Geography Standard [59] was used to classify each childcare service’s geographic location as metropolitan or regional, based on proximity to a major city. Area-level socioeconomic position (SEP) for each childcare service was determined using the Index of Relative Socioeconomic Advantage and Disadvantage [60]. Each service was allocated a decile score based on its postcode to determine the relative level of advantage and disadvantage (1=greatest disadvantage and 10=greatest advantage) in the local area. Descriptive statistics were generated for the following: (1) childcare service characteristics including location (metropolitan or regional), type of management (private or community), area-level low SEP (score of 1-3), middle SEP (score of 4-7), or high SEP (score of 8-10) and *FoodChecker* use (yes, no, or unsure); (2) participant characteristics including role (director or cook), years of employment, educational attainment, and nutrition training; and (3) nominal (yes or no) data about *FoodChecker* usefulness and changes in confidence, learning, and the childcare menu.

#### Thematic Analysis

The Braun and Clarke [61] approach to inductive thematic analysis was used to explore menu planner perceptions of *FoodChecker*, as is consistent with the qualitative descriptive methodology [62]. Through an iterative process, an open coding technique was used to assign previously undefined codes to raw data extracts using NVivo. To minimize the risk of bias, a 10% sample of survey response files (n=6) was independently analyzed by 2 researchers (JVK and ACS), each of whom developed a preliminary coding framework. Differences in the coding frameworks were discussed until a consensus was reached. This verification process has been previously used in inductive thematic analysis of qualitative descriptive research [63]. Data extracted from the remaining survey response files were coded by 1 researcher (JVK). The codes were systematically categorized to determine common themes and their trends, patterns, and relationships. Through ongoing iteration and analysis, themes were reviewed and discussed with the research team, refined and named, and then rechecked to ensure that they accurately reflected coded extracts and raw data.

### Application of Theory

**Figure 4:**
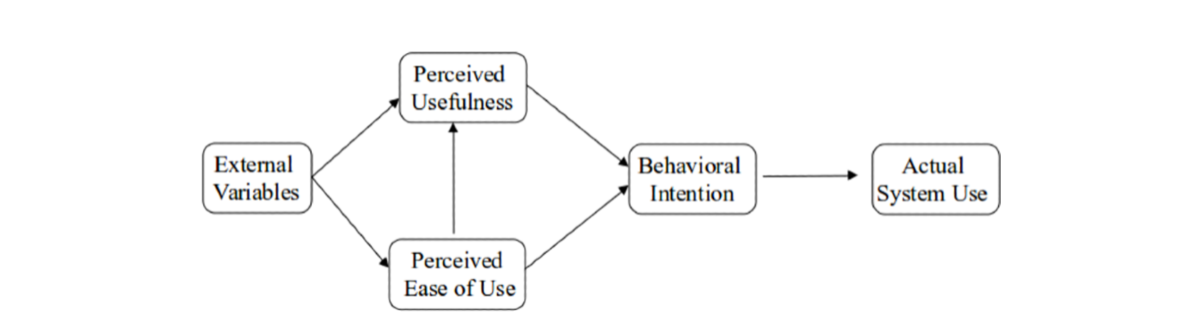
Technology Acceptance Model (Venkatesh and Davis [64]).

Constructs of the Technology Acceptance Model (TAM) were used to report themes identified from the inductive analysis related to the degree to which participants perceived *FoodChecker* would be useful and easy to use, and external variables specific to the individual or organization influencing perceptions and use (Figure 4 [64]). The TAM is a validated, widely used, and highly predictive model of information technology use [65], which posits that acceptance of a technology is directed by the degree to which users perceive the system to be useful (or enhance their job performance) and easy to use (or free from effort) [66]. It has been significantly associated with the intention to use a digital menu planning tool in the childcare sector [44].

## Results

### Participants

**Figure 5:**
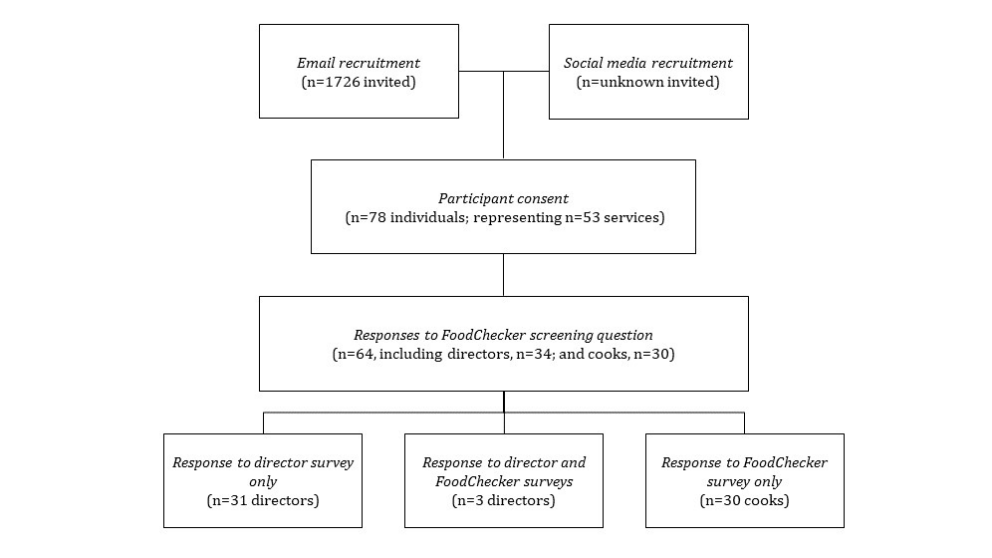
Childcare service and participant recruitment and survey respondents.

A total of 64 participants (comprising 34 directors and 30 cooks) from 53 childcare services participated in this study (Figure 5); 52% (33/64; n=30 cooks and 3 directors) responded to the *FoodChecker survey*. The remaining participants (31/64, 48% directors) responded to the *director survey* only.

### Childcare Service and Participant Characteristics

Childcare service and participant characteristics are presented in Table 2. Most services were located in metropolitan areas of Victoria (47/53, 89%) and privately owned (40/53, 75%). Half of the services (n=27) were located in regions classified as high SEP. Furthermore, 60% (32/53) of services reported using *FoodChecker* for menu planning. Most commonly, *FoodChecker* was used monthly or when menus were updated. Most participants had a minimum of certificate- or diploma-level qualification (55/64, 86%). Fewer than half (26/64, 41%) of the participants reported receiving nutrition training.

**Table 2:** Childcare service and participant characteristics.

Characteristics				Values, n (%)
**Childcare service (n=53)**				
	**Geographic location**			
		Metropolitan	47 (89)	
		Regional	6 (11)	
	**Management**			
		Private	40 (75)	
		Community	13 (25)	
	**Area-level SEP^a^**			
		Low SEP (scores 1-3)	10 (19)	
		Middle SEP (scores 4-7)	16 (30)	
		High SEP (scores 8-10)	27 (51)	
	***FoodChecker* use**			
		No	21 (40)	
		Yes	32 (60)	
	**Frequency of *FoodChecker* use (n=32)**			
		Once-off	3 (9)	
		Monthly	8 (25)	
		Every 3 months	4 (13)	
		Every 6 months	1 (3)	
		Whenever I update my menu	8 (25)	
		Other	1 (3)	
		No response	7 (22)	
**Participant (n=64)**				
	**Role**			
		Director	34 (53)	
		Cook	30 (47)	
	**Years of employment**			
		<1 year	7 (11)	
		1-2 years	6 (9)	
		2-3 years	10 (16)	
		3-4 years	3 (5)	
		>4 years	38 (59)	
	**Educational attainment**			
		≤Grade 12	3 (5)	
		Trade, apprenticeship, diploma, or certificate	34 (53)	
		University degree	21 (33)	
		No response	6 (9)	
	**Nutrition training**			
		Yes	26 (41)	
		No	32 (50)	
		No response	6 (9)	

^a^SEP: socioeconomic position.

### *FoodChecker* Usefulness and Changes in Confidence, Learning, and Menus

Participant responses to quantitative questions about *FoodChecker* are presented in Table 3. A total of 79% (26/33) of participants responded to quantitative questions about *FoodChecker*. Of these, the majority agreed *FoodChecker* was useful for their role (22/24, 92%) or childcare services (23/25, 92%). Furthermore, most participants agreed that due to using *FoodChecker*, their confidence about planning healthy menus had improved (20/26, 77%), they had learned something (17/24, 71%) and that their service’s menu had changed (17/25, 68%).

**Table 3:** Participant reports of *FoodChecker* usefulness and changes in confidence, learning, and menus (n=33)^a^.

Question	Response rate, n (%)	Yes, n (%)	No, n (%)
Do you think that online menu planning tools like *FoodChecker* are useful for childcare centers?	25 (76)	23 (92)	2 (8)
Do you think that online menu planning tools like *FoodChecker* are useful for your role?	24 (73)	22 (92)	2 (8)
As a result of using *FoodChecker*, has your confidence about planning healthy menus improved?	26 (79)	20 (77)	6 (23)
As a result of using *FoodChecker*, have you learnt something?	24 (73)	17 (71)	7 (29)
As a result of using *FoodChecker*, has your center's menu changed?	25 (76)	17 (68)	8 (32)

^a^Participant responses to the *FoodCheckersurvey* (30 cooks and 3 directors).

### *FoodChecker* Themes

#### Overview

From the thematic analysis, 10 common themes were constructed about *FoodChecker* use, which were reported according to TAM constructs (Table 4).

**Table 4:** Overview of 10 themes constructed from thematic analysis and organized under constructs of the Technology Acceptance Model (TAM).

TAM construct	Theme
Perceived usefulness	Theme 1: Supporting child nutrition and health Theme 2. Improving organizational processes Quality improvement and accountability Meeting food provision recommendations and standards Improving menu planning processes Improving menu quality Engaging families Theme 3. Aiding the menu planner Increasing confidence and learning Reducing workload Theme 4. Ways to improve usefulness
Perceived ease of use	Theme 5: Mixed perceptions about ease of use Theme 6: Ways to improve ease of use
External variables	Theme 7: Awareness and perceived need Theme 8: Time and resources Theme 9: Organizational support Theme 10: Food budget

### TAM Construct: Perceived Usefulness

Almost all participants, including directors and cooks, described *FoodChecker* as useful in their role or for the childcare sector, particularly for supporting child nutrition and health, improving organizational processes, and aiding the menu planner role.

#### Theme 1: Supporting Child Nutrition and Health

Child health was a priority for directors and cooks, who commonly discussed health motivations when describing the usefulness of *FoodChecker* in their role. This was demonstrated by one director who explained that the tool “changed the way we think about health” (director-34). Several cooks drew links between using *FoodChecker* and supporting children’s health. One explained that it helped their service provide “nutritious food to reduce illness” (cook-6) and another specified a “healthy diet has direct benefits to students’ mental health” (cook-13). Another drew a further link to children’s learning, stating that the web-based tool was useful “especially for us: Strong Foundations! Healthy eating and healthy bodies = learning!” (cook-3). Participants acknowledged that food provided at their service influenced children’s health and discussed using *FoodChecker* to ensure that menus met children’s nutrition and dietary needs. One cook stated, “it’s a part of the program we use to ensure our children have a balanced, nutritional diet” (cook-8). A director emphasized this was particularly important given “many children receive most of [their] meals/snacks at the service” (director-25).

#### Theme 2: Improving Organizational Processes

The overview of this theme has been described as follows:

1. *Quality improvement and accountability*: quality improvement was important for participants, as evidenced by a director who explained “anything that assists us in continuous improvement has great value” (director-20). Cooks and directors alike described the need for childcare services and staff to be accountable for food provision practices. For example, a director described using *FoodChecker* to “ensure menu planning is on track and give accountability to the chef” (director-23), while a cook discussed using it “to keep services accountable for what they feed their children. There are still far to[o] many services with terrible budgets and menus that are not well balanced” (cook-24).
2. *Meeting food provision recommendations and standards*: *FoodChecker* was considered useful for supporting services to meet food provision recommendations. For example, a cook described using the web-based system to ensure “best-practice nutritional guidelines for children are being met in daily menus...and make sure we are meeting all healthy eating standards for children” (cook-3). Another referred to national childcare standards, stating that meeting children’s daily nutrition needs is “required for rating and assessment” (cook-24). In some services, *FoodChecker* was used to meet food provision benchmarks within government-funded and endorsed health promotion initiatives. Indeed, one director stated, “As a part of the Achievement Program [67], we use *FoodChecker* to make sure our menus meet the daily intake of required foods and is healthy for all” (director-17).
3. *Improving menu planning processes*: several cooks expressed *FoodChecker* was valuable for improving menu planning processes. For example, they explained that *FoodChecker* “makes it easier and quicker to plan the menu” (cook-29) or “helps in the planning of meals, as it serves as a reference for appropriate quantities, number of serves, variety of food from the five food groups, and portion size... [it] also helps in wastage control, budgeting and ordering” (cook-5). *FoodChecker* was reported to offer further menu planning guidance through links to web-based resources including “healthy ingredient swaps and shopping tips” (cook-15) and ways “to deal with challenges such as allergies and budgeting” (cook-12).
4. *Improving menu quality*: most participants (17/25, 68%) who responded to the question about whether their childcare menu had changed because of using *FoodChecker* reported that menu changes had occurred owing to using the web-based program (Table 3). When describing these changes, some provided general information, explaining that they felt their menus were better, healthier, or more varied. For example, a director explained “we have a season[al] menu now where before it was a fortnightly menu which didn’t change” (director-17). Others described specific changes, such as adapting portion sizes or food provision (eg, providing more dairy, vegetables or grains, or less fatty, salty, or sweet foods). One cook explained that they had rearranged the menu so it was now “designed and implemented based on the recommendations available on the system” (cook-5). Some respondents also thought that menu changes had resulted in dietary changes, as one cook stated, “we eat and enjoy more nutritional foods” (cook-8).
5. *Engaging families*: for some participants, *FoodChecker* provided a platform for accessing recipes, information, and guidelines to share with families and support their engagement in menu planning. Indeed, a director explained “children have more input and... parents are asking for a copy of recipes... parents are using the menus at home” (director-17).

#### Theme 3: Aiding the Menu Planner

The overview of this theme is described as follows:

1. *Increasing confidence and learning*: most participants who responded to questions about whether *FoodChecker* had impacted their confidence and learning reported that using the web-based tool had helped them build confidence in menu planning (20/26, 77%) and learn information relevant to their role (17/24, 71%; Table 3). Several cooks explained that from the web-based system they “learned how to cook” (cook-21) or “how to plan food for the kids” (cook-20). One stated that they “learned a lot of nutrition knowledge, which is very useful” (cook-15).
2. *Reducing workload*: several cooks reported *FoodChecker* made it “easier and quicker” (cook-8) to plan menus and that using the web-based tool reduced their workload. Some explained “it lightened a lot of work and made me more relaxed” (cook-14) or “it lightens my workload. I’m very satisfied that I can do other things” (cook-16).

#### Theme 4: Ways to Improve Usefulness

Although there was agreement about the usefulness of *FoodChecker*, the participants described updates that would further improve its value. For example, a cook recommended “Keep improving the tool... Provide us with accreditation evidence (a tick) so that families can see that our menus meet *FoodChecker* standards” (cook-3). Others suggested that the tool should provide information about food suppliers. Further recommendations were provided regarding additional resources that could be made available through the platform, such as sample menus, ingredient substitutions, nutrition information, allergy resources, and a greater variety of recipes. One cook noted the need for the digital system to ensure confidentiality, stating “I also need to know my recipes are private and won’t be used in any way without my authorization” (cook-24).

### TAM Construct: Perceived Ease of Use

#### Theme 5: Mixed Perceptions About Ease of Use

Perceptions about the ease of use of *FoodChecker* were mixed. Some participants reported that the tool was “easy to use” (director-23) or noted specific elements such as an “easy checklist to ensure a balanced weekly menu” (cook-3). Conversely, some described the web-based functionality as “not very easy to navigate” (director-13) or “a little too complicated in some ways” (cook-21). One cook stated it was “...too confusing, I needed help...computers are tricky for me... I don’t like to use [it]” (cook-7).

#### Theme 6: Ways to Improve Ease of Use

Some participants stated that updates to navigation and functionality within the tool would make it easier to use. One cook, who described *FoodChecker* to be useful in their role, also stated “I just hope they work on the navigation of the site” (cook-17). Others discussed the need to integrate strategies to reduce data input time, explaining that “data input is quite time-consuming” (cook-5) or suggested the need for functionality to easily fix errors in data input, for example, “not having to start the entire menu over for small incorrect servings” (cook-4).

### TAM Construct: External Variables

Participants identified several variables that influenced their perceptions of *FoodChecker* and their use of the tool.

#### Theme 7: Awareness and Perceived Need

Some participants had not used *FoodChecker* because they were unaware of the tool. One cook explained “I didn’t know about it until now. I have registered and will look at it from now on” (cook-2). Others who did not use *FoodChecker* perceived their service as having adequate processes in place to ensure healthy menu planning. This included the presence of cooks and directors believed to be adequately skilled in healthy menu planning, as well as input from staff, parents, and children. One director explained “we are a small private center with a self-managed system in place that works well” (director-21). Others accessed support from external consultants or used “the alternative [menu planning tool] from *feedAustralia*” (director-5).

#### Theme 8: Time and Resources

Time was described as an important factor related to *FoodChecker* use. Participants commonly stated that inputting menu data into the program was “very time consuming” (director-13). The short turnaround times for planning new menu cycles and the need to reassess menus with each change presented challenges. For example, participants explained that they “normally allow two weeks to complete [a] new season menu” (director-17) and “each time we change a menu, we need to food check again” (cook-3).

Lack of time was a reported barrier to *FoodChecker* use, as a director explained, “the cook is aware of *FoodChecker* but is limited on time to use this service” (director-14). For one cook, lack of time was exacerbated by a lack of technological resources, as they explained, “there is no computer or iPad in the kitchen” (cook-24). They further described the challenge of competing priorities within their role, stating they:

Get menu planning time, [b]ut it is also documentation time, cleaning, and food safety plan time. As well as newsletters and posting on story park. So [I] need to prioritize the work and *FoodChecker* is sometimes last.

Cook-24

Others reported that dedicated “paid time for menu planning” (cook-4) within their role enabled them to use *FoodChecker*.

#### Theme 9: Organizational Support

Cooks reported that management support and leadership facilitated their use of *FoodChecker*. One cook expressed they received “encouragement, time and practical support from management to use *FoodChecker*” (cook-3) and another explained, “the director and teachers of our center are very satisfied and give us the greatest support” (cook-15). In some services, using the web-based system was perceived to be a directive from management. Cooks discussed using *FoodChecker* at the “request of the business to ensure that we meet (and exceed) the nutritional requirements of the children” (cook-5) or that it was “part of our policy...to use *FoodChecker*” (cook-29).

#### Theme 10: Food Budget

The food budget was an important factor for cooks, as reported by one participant who stated, “cost control is our biggest headache” (cook-15). While some reported *FoodChecker* provided links to web-based resources that supported their service with budgeting, others explained that recommendations made by the *FoodChecker* system presented challenges for the food budget. For example, they explained that when using *FoodChecker* they were “unable to control costs” (cook-12) or that it was “easy to exceed our budget and buy food materials” (cook-16).

## Discussion

### Principal Findings

In this novel study, we aimed to explore the use of a web-based menu planning tool for childcare services. Among the first of its kind, the study sought insights and perspectives from childcare cooks and directors, in their own words, about their use of a web-based tool to plan menus and guide food provision for children in childcare. The study found that cooks and directors alike considered the web-based tool to be useful in their roles, although use was influenced by a variety of factors including awareness, perceived need, time, resources, organizational support, and budgetary considerations. Participants made recommendations to improve the web-based tool, including the need to update navigation and functionality, integrate strategies to reduce data input time, and provide more links to relevant web-based resources to support healthy menu planning.

### Comparison With Prior Work

To the best of our knowledge, this is the first study to consider insights from both cooks and directors about their use of a web-based childcare menu planning tool, with limited previous analyses focusing on acceptance by childcare directors only [42,43]. As such, this study offers an end-user perspective most likely to represent insights from both staff groups involved in menu planning. Participants described a variety of motivations important for menu planning, particularly the need to support children’s health and nutrition, improve food provision and menu planning processes, and aid the menu planner role. Similar motivations have been reported in previous analyses of childcare menu planners [68,69]. However, this is the first study to document the usefulness of a web-based menu planning tool for integrating these motivations into practice.

In this study, directors and cooks emphasized the value of a web-based menu planning tool for both the childcare sector and within their specific role. Comparatively, in limited prior evidence, reports on the perceived value of web-based menu planning tools in childcare settings have been mixed. For instance, while directors have previously reported high intentions to use a web-based menu planning tool and high levels of computer access [44], cooks have reported not using or requiring web-based menu planning tools and having limited computer literacy and access [68]. This discordance could indicate differing needs, levels of computer access, or perceived levels of computer literacy between cooks and directors in childcare settings. Indeed, in this study, one cook reported low levels of digital literacy, and another indicated that a lack of technology in the kitchen was a barrier to using a web-based menu planning tool. These challenges were not reported by the directors.

In this study, the web-based menu planning tool was considered valuable for supporting engagement with families, particularly for sharing menus and recipes. This is important, given that family engagement is widely recommended to increase the impact of childcare-based healthy eating interventions [70]. Most childcare services use web-based platforms to communicate with families [44], indicating the potential to extend the use and reach of web-based menu planning tools to the family and home environment. Indeed, this is demonstrated in the *feedAustralia* intervention where parents can use a mobile app to view daily food offerings and access sample menus and recipes that they can recreate at home [71].

Despite the consensus about the usefulness of the web-based menu planning tool in this study, there were mixed reports from both directors and cooks about how easy it was to use the tool in practice. It stands to reason that user training may improve ease of use, as reported in the *Go-NAPSACC* trial [42]. However, the participants in this study did not discuss the need for *FoodChecker* training but rather the need to update navigation and functionality within the web-based tool to make it easier to use. Given that up to 80% of health technologies have limited success owing to a lack of end-user adoption or sustained use [45], understanding and integrating user preferences within web-based systems is necessary to increase their use and impact [72]. Future developments of web-based childcare menu planning tools should therefore consider strategies to improve user engagement (such as simpler site navigation or functionality to facilitate faster data input), to amplify their adoption, for sustained use over time, and for public health impact. This indicates the importance of directing funding toward the ongoing development of web-based menu planning tools for the childcare sector to meet user expectations, particularly in the current era of rapid technological advancement.

The limited evidence available has demonstrated that, even when the acceptability of a web-based menu planning program in the childcare sector is high, use may still be variable [42,43]. This indicates that factors external to the system itself (eg, individual or organizational factors) may influence use. Exploration of such factors was novel to this study, with participants reporting that awareness, perceived need, time, management support, and the food budget were important variables relevant to their uptake of the web-based menu planning tool.

Several participants were unaware of the tool and, as such, had not used it. Others who were aware of the tool but did not use it perceived that childcare staff had adequate nutrition knowledge and skills to plan a healthy menu. Given the scope of this study did not include a menu assessment component, it was not possible to triangulate staff perceptions about their knowledge and skills in healthy menu planning and the degree to which childcare menus complied with food provision guidelines. This is an important direction for future research. Despite this, there were multiple instances in which both cooks and directors demonstrated good knowledge of the scientific evidence related to their roles. For example, participants discussed the need to provide optimum nutrition to children who received most of their meals and snacks in childcare or links between healthier eating and children’s health outcomes, as evidenced in the literature [1,24]. Although these examples of accurate nutrition knowledge are reassuring, it cannot be inferred that this knowledge is consistent or accurately translated into menu planning practices, indicating the need for healthy menu planning support in the sector.

Although support in the form of nutrition training has been shown to improve menu quality, such training is not routine [25]. Indeed, less than half of the participants in this study reported having received nutrition training. This is similar to previous studies where childcare staff reported low levels of nutrition training despite their responsibility to plan a healthy menu that meets food provision guidelines [25]. This indicates that there is scope for the design and implementation of interventions, such as web-based menu planning tools that offer accessible, evidence-based guidance that can be integrated into menu planning practices, even in the absence of formal staff nutrition training.

Some participants who stated that they did not use the web-based menu planning tool reported using an external consultant to support healthy menu planning in their service. Such consultancy may increase the financial burden of healthy menu planning, and may not be viable for all childcare services. However, it may also present a sensible approach to ensure accuracy in menu compliance with dietary guideline recommendations, if such support is available at a feasible cost.

Time was an important factor in this study, with mixed perceptions among the participants. Staff members who received dedicated and paid time within their role to use the web-based menu planning tool reported that this facilitated its use. Moreover, some participants who used the tool reported that it was time saving and reduced their workload. However, as in previous studies [73], most participants did not report receiving paid time for menu planning within their roles. Furthermore, the use of the tool was commonly perceived to be time consuming, even by participants who had never used it before. As such, perceptions about time were a deterrent to initial adoption. This indicates that there is scope to (1) integrate user-engagement features within childcare menu planning systems to improve time efficiency and (2) establish strategies to shift user perceptions about such systems from being *time consuming* to being *time saving*. These are important areas for future development, particularly given that childcare staff members are known to be a time-poor population group [36].

In addition to dedicated time, service management support and leadership were reported to facilitate the uptake of the web-based menu planning tool. Interestingly, one cook who did not want to use the program had used it on instruction from their manager to ensure menu compliance with dietary guidelines. This indicates that despite the presence of management support, menu planner resistance may exist and could be a potential barrier to ongoing use of the web-based menu planning tool.

The food budget and cost control were important considerations for participants who described budgeting resources linked to the web-based menu planning tool as useful. However, several cooks reported that specific food recommendations generated by the web-based system exceeded their allocated food budget. This is a novel finding, indicating the need for dietary guidelines and recommendations embedded within such systems to consider food budgets and financial constraints. It may also suggest the need to investigate the capacity of childcare food budgets to adequately provide optimal nutrition for children. There is scope for web-based childcare menu planning systems to integrate strategies to support services in establishing and managing food budgets, which is an area for future research and development.

Although the services in this study were located in areas of varying levels of advantage and disadvantage, half were located in regions classified by postcode as high SEP. This is consistent with data indicating that, in Victoria, there is a higher provision of childcare services in areas experiencing greater levels of advantage [23]. However, as the study achieved a modest sample size, it was not possible to determine whether area-level SEP was associated with the acceptance of the web-based menu planning tool or the degree to which the tool supported healthy food provision in childcare services. Given that area-level disadvantage is associated with poorer diet quality [74], and given that services in low SEP areas were least represented in this study, there is a need for future research to better understand the use of web-based childcare menu planning tools in lower SEP areas.

### Strengths and Limitations

The strengths of this study warrant discussion. As this study captured perspectives from cooks and directors, the findings are likely to represent the perspectives of both staff groups involved in menu planning. The study’s qualitative descriptive underpinning provided scope to capture participant-generated data on menu planner experiences in their own words. Insights generated from spontaneous reporting were likely to reflect motivations and perspectives that are most important to users when compared with quantitative analyses using predefined, researcher-generated items and response scales [62]. The use of the validated TAM contributed to the study’s underlying theoretical foundation. In line with emerging methodology-focused evidence, a web-based qualitative survey facilitated data collection from a time-poor and varied, dispersed, and geographically heterogeneous population with the potential to reduce social desirability bias [50]. A further strength of this study was that it analyzed the use of a real-world web-based tool in current practice, to which real-time refinements are possible. As such, the exploration brought together research, reflection, and practical solutions as part of an action research approach.

Given that the *FoodChecker* tool is only freely available in Victoria, the scope of this study was purposely delimited to Victorian childcare services. A modest study sample was achieved, as observed in previous studies on food provision in childcare settings [68]. Furthermore, given that childcare staff are known to be a time-poor population group, the perspectives of those who have particularly limited time to engage with a web-based menu planning tool, and as such, to participate in this research, may not have been captured. This may impact the ability to generalize the findings to the broader childcare sector. However, as the analysis explored participant perceptions and insights that are subjective by nature, the findings may be indicative of childcare menu planner experiences more broadly. This should be further investigated in larger studies exploring the use of web-based menu planning tools to support healthy food provision in childcare. It was beyond the scope of this study to include a quantitative analysis of the impact of the web-based menu planning tool on menu compliance with dietary guidelines, food provision, or children’s dietary intake. These are important areas for future research.

### Conclusions

This novel qualitative descriptive study demonstrates the usefulness of a web-based tool to support healthy menu planning in childcare services. Use of the tool was impacted by its internal functionality as well as external organizational factors. Recommendations were made to improve the web-based menu planning system.

Further research is needed to better understand how web-based menu planning tools can improve food provision and children’s consumption in the childcare setting. In particular, studies should investigate and evaluate strategies to improve user engagement with web-based menu planning tools in childcare to increase their adoption, use, and public health impact.

## Abbreviations

NAPSACC: Nutrition and Physical Activity Self-Assessment for Child Care
NAV: Nutrition Australia Victorian Division
RCT: randomized controlled trial
REDCap: Research Electronic Data Capture
SEP: socioeconomic position
TAM: Technology Acceptance Model
